# The antisense protein of HTLV-2 positively modulates HIV-1 replication

**DOI:** 10.1186/1742-4690-11-S1-P118

**Published:** 2014-01-07

**Authors:** Cynthia Torresilla, Sonia Do Carmo, Émilie Larocque, Estelle Douceron, Jean-Michel Mesnard, Renaud Mahieux, Benoit Barbeau

**Affiliations:** 1 Département des sciences biologiques and Centre de recherche BioMed, Université du Québec à Montréal, Montréal (Québec) Canada; 2 Oncogenèse Rétrovirale, Ligue Nationale Contre Le Cancer, CIRI, INSERM U1111-CNRS UMR5308, Université Lyon 1, Ecole Normale Supérieure de Lyon, LabEx ECOFECT, Lyon, Cedex 07, France; 3 Université Montpellier 1, Centre d’études d’agents Pathogènes et Biotechnologies pour la Santé, CNRS, UM5236, Montpellier, France

## 

Unlike HTLV-1, HTLV-2 does not induce leukemia and has been tentatively associated with an HTLV-1-associated myelopathy-like disorder. It has been reported that HTLV-2/HIV-1 co-infected patients progress less rapidly to AIDS than HIV-1-infected individuals. Tax2 has been suggested to mediate this protective state by inducing MIP-1α expression and blocking HIV-1 infection. As cells from HTLV-2-infected individuals mainly express Antisense Protein 2 (APH-2), our objective was to determine if this protein might also intervene in controlling HIV-1 replication in dually infected individuals. Using Jurkat cells, we first demonstrated that both APH-2 and HBZ, the HTLV-1 analogue, equally induced MIP-1α in unstimulated and stimulated Jurkat T cells. To assess if APH-2 might directly affect HIV-1 replication, a full length luciferase-expressing proviral DNA was tested in Jurkat cells. Surprisingly, upon co-transfection with an APH-2 expression vector, an increase in luciferase activity was observed, while HBZ expression rather led to reduced reporter gene expression. Western blot analyses and ELISA assay further indicated that HIV-1 p24 levels were more important in APH-2-expressing cells. To determine if APH-2 was directly modulating HIV-1 LTR activity, both NF-κB and NFAT were tested in stimulated Jurkat cells. Unexpectedly, HBZ and APH-2 inhibited NF-κB and NFAT activation, albeit at different extent. In addition, LTR activation was also inhibited by both antisense proteins although APH-2 had a more modest effect. Our results thus highlight the complex interplay between HTLV antisense transcript-encoded proteins and HIV-1 expression and further studies will be required to determine the potential impact of APH-2 in HTLV-2/HIV-1-infected individuals.

